# Cytoneme-Mediated Delivery of Hedgehog Regulates the Expression of Bone Morphogenetic Proteins to Maintain Germline Stem Cells in *Drosophila*

**DOI:** 10.1371/journal.pbio.1001298

**Published:** 2012-04-03

**Authors:** Patricia Rojas-Ríos, Isabel Guerrero, Acaimo González-Reyes

**Affiliations:** 1 Centro Andaluz de Biología del Desarrollo, CSIC/Universidad Pablo de Olavide, Sevilla, Spain; 2 Centro de Biología Molecular Severo Ochoa, CSIC/Universidad Autónoma de Madrid, Madrid, Spain; University of California, San Francisco, United States of America

## Abstract

Stem cells reside in specialised microenvironments, or niches, which often contain support cells that control stem cell maintenance and proliferation. Hedgehog (Hh) proteins mediate homeostasis in several adult niches, but a detailed understanding of Hh signalling in stem cell regulation is lacking. Studying the *Drosophila* female germline stem cell (GSC) niche, we show that Hh acts as a critical juxtacrine signal to maintain the normal GSC population of the ovary. Hh production in cap cells, a type of niche support cells, is regulated by the Engrailed transcription factor. Hh is then secreted to a second, adjacent population of niche cells, the escort cells, where it activates transcription of the GSC essential factors Decapentaplegic (Dpp) and Glass bottom boat (Gbb). In wild-type niches, Hh protein decorates short filopodia that originate in the support cap cells and that are functionally relevant, as they are required to transduce the Hh pathway in the escort cells and to maintain a normal population of GSCs. These filopodia, reminiscent of wing disc cytonemes, grow several fold in length if Hh signalling is impaired within the niche. Because these long cytonemes project directionally towards the signalling-deficient region, cap cells sense and react to the strength of Hh pathway transduction in the niche. Thus, the GSC niche responds to insufficient Hh signalling by increasing the range of Hh spreading. Although the signal(s) perceived by the cap cells and the receptor(s) involved are still unknown, our results emphasise the integration of signals necessary to maintain a functional niche and the plasticity of cellular niches to respond to challenging physiological conditions.

## Abstract 2

Genetic manipulation of the germline stem cell niche in *Drosophila* ovaries reveals that support cells ensure the maintenance of stem cells by modulating the spread of Hedgehog within the niche.

## Author Summary

The *Drosophila* ovary contains a well-defined stem cell niche that hosts 2–3 germline stem cells (GSCs). The Hedgehog (Hh) family of signalling proteins mediates cellular homeostasis in several adult tissues, and here we decipher the detailed mechanism of action of Hh in the adult female GSC niche. We demonstrate that Hh acts in a juxtacrine manner (i.e., it requires physical contact between the cells involved) to maintain the normal pool of GSCs in the ovarian niche. Hh is produced in one type of niche support cell (the cap cells), and it is received, upon secretion, by a second, neighbouring population of niche cells (the escort cells). In the latter, we show that the Hh signalling pathway regulates the expression of the *Drosophila* Bone Morphogenetic Protein (BMP) homologues and essential stem cell factors *decapentaplegic* (*dpp*) and *glass bottom boat* (*gbb*). We also find that Hh distribution in the GSC niche is mediated by short cellular projections, reminiscent of wing disc cytonemes, although they grow from the (Hh) signal-producing cells towards the receiving cells. Under conditions of low levels of Hh protein and/or Hh signalling within the niche, cap cells emit up to 6-fold longer Hh-decorated cytonemes towards the signalling-deficient area of the niche. Our data reveal that stem cell niches are dynamic structures that can sense, and react to, changes in the activity of essential stem cell factors to prevent stem cell differentiation.

## Introduction

Stem cells are responsible for the integrity of tissues during growth, ageing, and repair. They reside in specialised microenvironments, or niches, which frequently comprise support cells that control stem cell self-renewal, proliferation, and differentiation [1],[2]. Stem cell niche regulation often involves short-range signalling between stem cells themselves and the surrounding microenvironment. One such short-range signal is the Hedgehog (Hh) family of proteins, which mediates homeostasis in several adult tissues, including the gastrointestinal tract, the hematopoietic system, and the vertebrate central nervous system [3]–[7]. In fact, Hh signalling dysfunction can lead to stem cell depletion or proliferative disorders such as tumourigenesis [8],[9]. However, the detailed mechanisms by which Hh acts in stem cell maintenance remain elusive.

**Figure 1:**
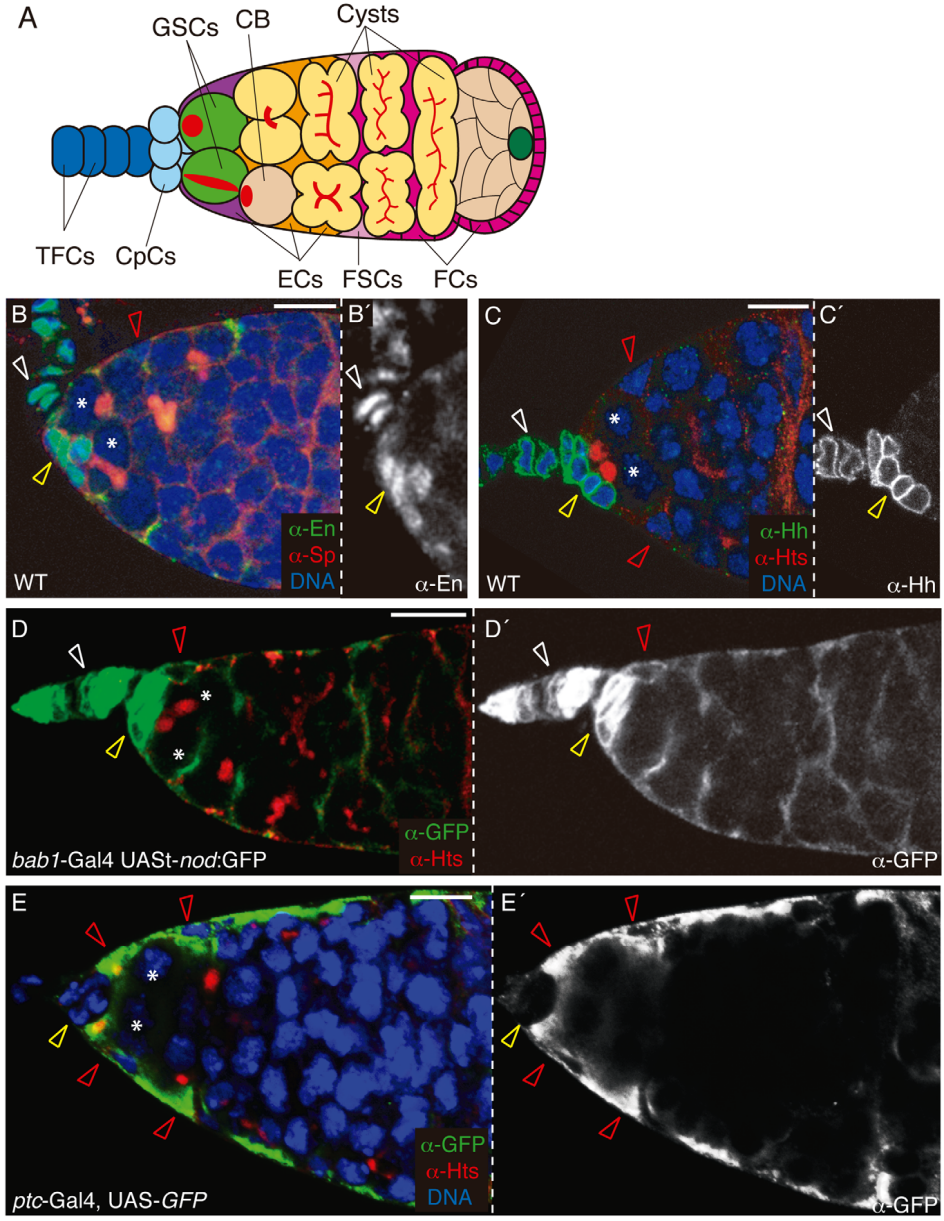
*en* and *hh* are expressed in TFCs and CpCs in the ovarian niche. (A) Schematic diagram of a germarium showing the support cell types (namely TFCs, CpCs, and ECs) and the germline cells (including GSCs, cystoblasts [CB], and developing cysts). GSCs are surrounded by 1–2 ECs and they contain an apical organelle called a spectrosome (red), which adopts an elongated shape after GSC asymmetric division. Cystoblasts also contain spectrosomes, but these are localised randomly within the cell. Cystoblasts undergo four incomplete rounds of division to give rise to 2-, 4-, 8-, and 16-cell cysts interconnected by branched fusomes (red). FCs, follicle cells; FSCs, follicle stem cells. (B) Wild-type (WT) germarium triple stained to visualise the expression of En in TFCs and CpCs (green), α-Spectrin in spectrosomes and fusomes (α-Sp; red), and DNA (blue). (C) Wild-type germarium stained with anti-Hh to visualise TFCs and CpCs (green), anti-Hts to label spectrosomes and fusomes (red), and Hoechst (for DNA; blue). (D) *bab1*-Gal4 UASt-*nod*:GFP germarium stained with anti-GFP to label TFCs, CpCs, and ECs (albeit with a weaker staining; green) and anti-Hts (red). (E) *ptc*-Gal4, UAS-GFP germarium stained with anti-GFP to label ECs (green), anti-Hts (red), and Hoechst (blue). Asterisks, GSCs; white open arrowheads, wild-type TFCs; yellow open arrowheads, wild-type CpCs; red open arrowheads, wild-type ECs. Scale bars: 10 µm.

In *Drosophila* females, germline stem cells (GSCs) are located at the apex of the ovary, in a structure termed the germarium that constitutes a well-defined stem cell niche. The germarium hosts three types of somatic niche cells: terminal filament cells (TFCs), cap cells (CpCs), and escort cells (ECs), which support two to three GSCs and which can be labelled with specific markers such as the *bab1*-Gal4 and *patched*-Gal4 drivers (Figure 1) [10]. The spatial organisation of the GSC niche permits direct contact between two to three CpCs and one GSC, which is anchored to the CpCs by adherens junctions [11]. In addition, approximately two ECs almost completely surround a given GSC [12]. The coordinated action of GSCs and their support cells allows continuous egg production during adulthood. Thus, GSCs normally divide asymmetrically to produce a differentiating cystoblast and a lineage-renewing GSC daughter [13]. Cystoblasts divide four times to give rise to 2-, 4-, 8-, and 16-cell cysts. ECs transfer the differentiating germline cystoblasts and cysts down the germarium using dynamic cytoplasmic processes [14],[15]. Germline cells in the germarium contain specialised organelles rich in membrane skeletal proteins that adopt a spherical (called spectrosome) appearance in GSCs and cystoblasts. Upon germline differentiation, the spectrosome grows in size and becomes a branched structure, termed fusome, characteristic of differentiating cysts. Hence, GSCs can be unambiguously identified by their location within the niche (in direct contact with CpCs) and by the presence of spectrosomes (Figure 1).

Reciprocal crosstalk between GSCs and support cells shapes the niche. Firstly, the size and organisation of the CpC cluster depends on proper Notch signalling between GSCs and CpCs [16]. Secondly, both the CpCs and the adjacent ECs play an important role in GSC maintenance, as they transduce the Janus kinase/Signal transducer and activator of transcription (Jak/Stat) pathway to induce the production of the Bone Morphogenetic Protein (BMP) protein Decapentaplegic (Dpp) [12],[17],[18]. Thirdly, the germline lineage activates the epidermal growth factor receptor pathway in the ECs to repress *dally* expression, thus limiting Dpp movement and stability [19]. Because Dpp (and another BMP homologue called Glass bottom boat [Gbb]) [20],[21] act directly on GSCs to repress differentiation and promote self-renewal [22],[23], the control of BMP activity is of the utmost importance for correct GSC niche homeostasis.

Here, we demonstrate a key role for the Hh pathway in the regulation of BMP signalling in the *Drosophila* female GSC niche. In addition, we found that wild-type niche support cells grow short Hh-coated filopodia that are functionally relevant for GSC maintenance. Furthermore, support cells sense dysfunctional Hh signalling within the niche and react by growing up to 6-fold longer cytonemes that help increase the range of Hh ligand spreading.

## Results

### *engrailed* Is Required Specifically in CpCs for GSC Maintenance

**Figure 2:**
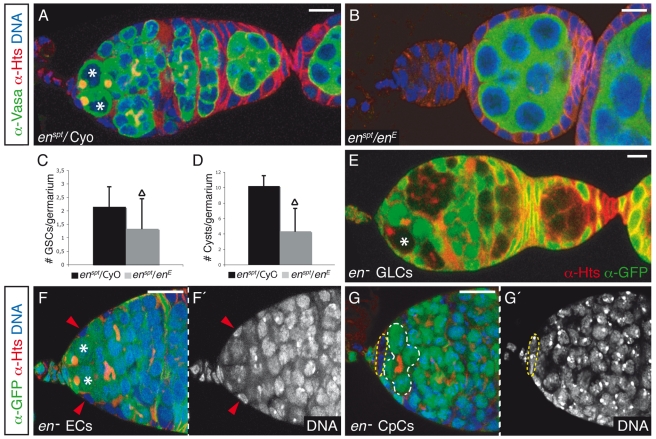
*en* controls germline development. (A and B) Control (A) and experimental (B) germaria dissected after 7 d at restrictive temperature (28°C). While the control germarium shows two GSCs and several differentiating cysts, the mutant is devoid of germline cells. (C and D) Bar chart representations of the mean number of (C) GSCs (± standard deviation [s.d.]) and (D) cytoblasts and developing cysts (± s.d.) per germarium in control and experimental germaria. Triangles indicate statistically significant differences (Student's *t* test, *p*<0.0005). (E) *en* ^E^ germline clones dissected 21 d after heat shock. *en* is not required in the germline. (F) *en* ^E^ EC clones do not affect GSC maintenance. (G) *en* ^E^ CpCs induce GSC differentiation as shown by the appearance of branched fusomes adjacent to the mutant CpCs (see also Figure S2 and Table S1). Somatic clones were induced with the *bab1*-Gal4 driver and were dissected 3 d after eclosion. Asterisks, GSCs; GLCs, germline clones; yellow dashed lines, *en* mutant CpCs; white dashed line, four-cell cyst; red arrowheads, *en* mutant ECs. Scale bars: 10 µm.

In a number of tissues, the Engrailed (En) transcription factor regulates *hh* expression. Because both *en* and *hh* are expressed in TFCs and CpCs (Figure 1B and 1C), and considering the importance of the Hh signalling cascade in stem cell maintenance in insects and vertebrates [24],[25], we tested whether the *en/hh* connection played a role in the GSC niche. To generate *en*-deficient germaria, we cultured adult females bearing a thermosensitive *en* allele (*en* ^spt^) in combination with an *en* deficiency (*en* ^E^) for 7 or 14 d at restrictive temperature (28°C; hereafter referred to as *en* ^ts^ germaria). Compared to control germaria (*en* ^spt^ */CyO*), which contained an average of 2.3±0.8 GSCs and 10.2±1.3 developing cysts (*n* = 62; 7 d) and 2.1±0.9 GSCs and 9.1±3.1 developing cysts (*n* = 49; 14 d), *en* ^ts^ germaria showed a significant decrease in the average number of GSCs and cysts (1.4±1.1 GSCs and 4.3±2.9 developing cysts, *n* = 52, 7 d; 1.2±0.8 GSCs and 3.9±2.5 developing cysts, *n* = 41, 14 d). Interestingly, 28.6% of *en* ^ts^ germaria analysed after 7 d at restrictive temperature were devoid of germline cells, which emphasised the importance of *en* gene function in GSC maintenance (Figure 2A–2D; Table S1). To distinguish between a requirement for *en* in the germline versus in the niche support cells, we abolished *en* function from either GSCs or niche cells by utilising two genetically null alleles, *en* ^E^ and *en* ^54^. The removal of *en* from the germline did not affect oogenesis, even 3 wk after gene inactivation (*n*>30 for each genotype; Figure 2E). To eliminate the activity of *en* in TFs, CpCs, or ECs we utilised the *bab1*-Gal4 driver (Figure 1D). Similar to the removal of *en* from the germline, elimination of *en* from all ECs in contact with a given GSC did not yield a visible phenotype (100% of cases, *n* = 23; Figure 2F). However, in 67.7% (*n* = 37) of mosaic germaria where *en* function was removed from at least three clustered CpCs, we observed differentiating cysts that contained branched fusomes and showed the accumulation of the differentiation marker Orb in contact with CpCs (Figures 2G and S1), a phenotype never found in wild-type germaria. Because we did not detect increased apoptosis in mosaic germaria contaning *en* mutant CpCs and since these mutant cells still expressed CpC markers (Figures S1 and S2), we conclude that *en* is required in CpCs to prevent GSC differentiation.

### Hh Release from CpCs Is Required for GSC Maintenance

**Figure 3:**
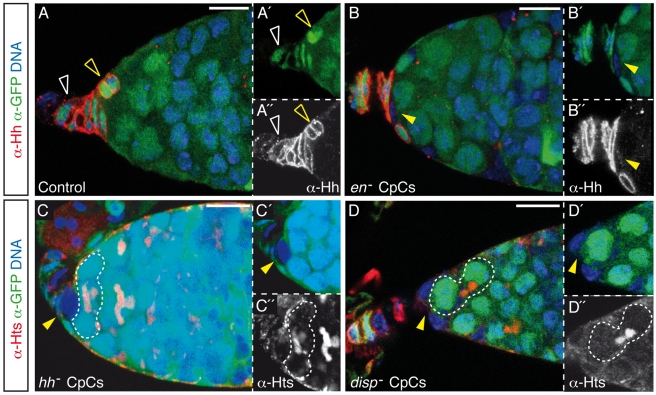
*en* controls Hh protein levels and Hh secretion is required for GSC maintenance. (A) Control germarium. Hh is expressed specifically in TFCs and CpCs. (B) *en* ^E^ mutant CpC showing a strong decrease in Hh levels. (C and D) *hh* ^AC^ (C) or *disp* ^SH21^ (D) mutant CpCs can induce GSC differentiation, as visualised by the presence of differentiating cysts adjacent to CpCs. See also Figure S3 and Table S2. Ovaries of the appropriate genotypes were dissected 3 d after eclosion. White open arrowheads, wild-type TFCs; yellow open arrowheads, wild-type CpCs; yellow arrowheads, mutant CpCs; white dashed lines, differentiating cysts in contact with CpCs. Scale bars: 10 µm.

The effect on the germline of removal of En from CpCs suggested the existence of one or more En-dependent niche cell signals that act on GSCs to promote their maintenance. Hh expression in TFCs and CpCs has been shown to be required for germline development [26] (Figures 1 and S3), which made Hh an excellent candidate to mediate En function in GSC maintenance. We examined the distribution of Hh in mosaic germaria that contained *en* mutant cells and found that *en* was required in a cell-autonomous fashion for strong membrane accumulation of Hh in TFCs and CpCs (81.8% of mutant cells, *n* = 98; Figure 3A and 3B). In addition, we established that the removal of Hh from at least three adjacent CpCs induced GSC differentiation (51.3% of cases, *n* = 39; Figure 3C). It has been shown that the release of the cholesterol-modified form of Hh requires the activity of the *dispatched* (*disp*) gene [27]. Interestingly, we found that the removal of *disp* from CpCs was also associated with the appearance of differentiating cysts within the mosaic niche, albeit at a lower frequency (31.6% of germaria with clusters of ≥3 mutant CpCs, *n* = 19; Figure 3D).

The incomplete penetrance of GSC differentiation in *en* and particularly in *hh* or *disp* mosaic niches was most likely due to non-autonomous Hh release from the remaining wild-type cells present in the niche. In fact, the larger the number of *hh* mutant CpCs, the fewer GSCs remained in the niche (see below and Table S2). Alternatively (or in addition), *disp* mutant CpCs may still be able to sustain a certain level of Hh signalling to adjacent ECs, as shown for the wing disc [27],[28]. Because the absence of either *hh* or *disp* from other niche cells, such as TFCs or ECs, did not cause a visible GSC phenotype (data not shown), and considering the requirement for Disp in cholesterol-modified-Hh release, these results strongly suggest that Hh needs to be produced in, and secreted from, CpCs to support a stable GSC population.

### Activation of the Hh Pathway in ECs Prevents GSC Differentiation

**Figure 4:**
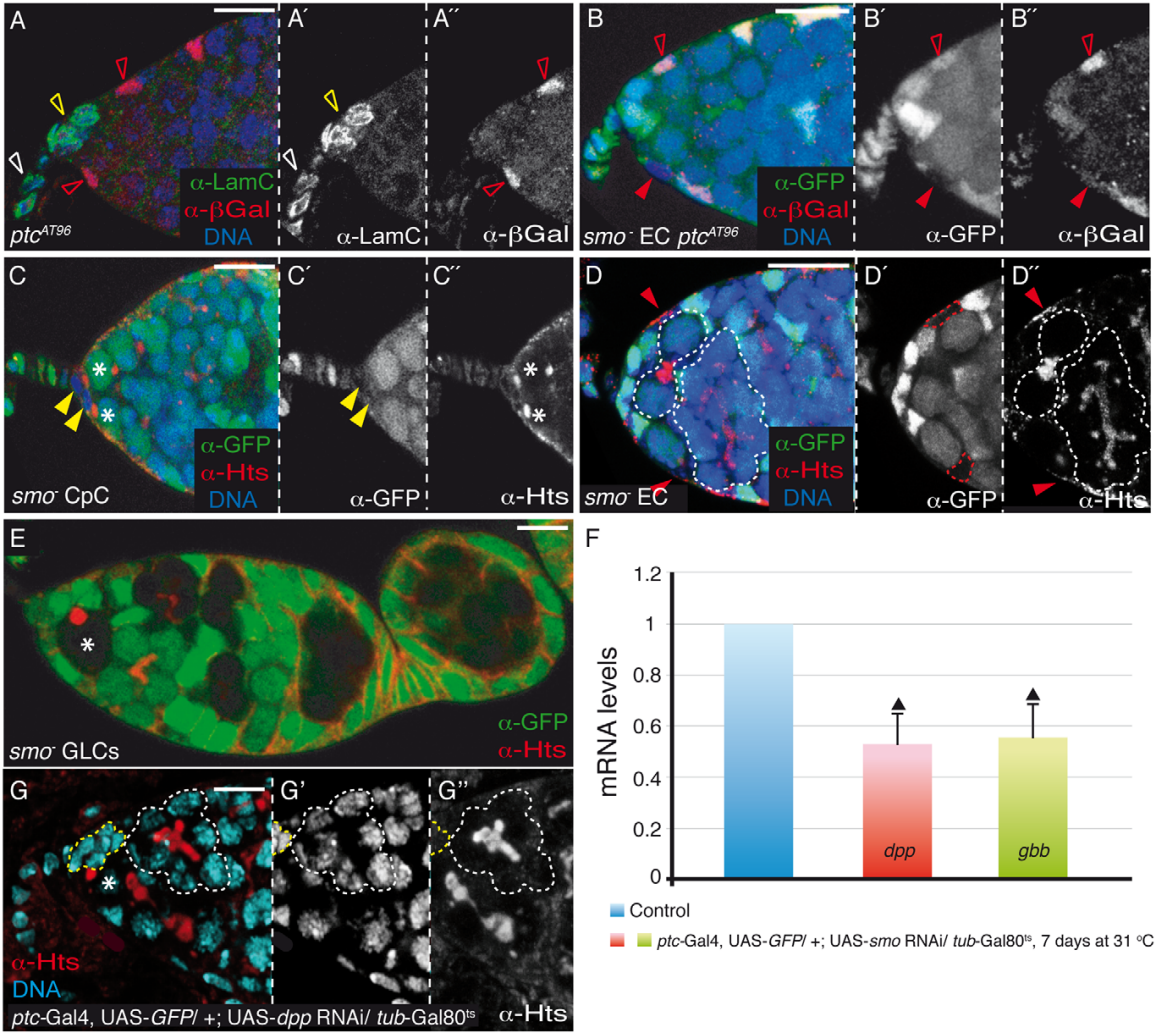
The Hh signalling pathway is required in ECs for GSC maintenance. (A) *ptc* ^AT96^ (*ptc*-lacZ) germarium stained to visualise lacZ expression in ECs. Lamin C (LamC) is a marker of TFCs and CpCs. (B) *smo* ^3^ mutant EC showing an undetectable expression of *ptc*-lacZ beyond background levels. (C) *smo* ^D16^ mutant CpCs do not induce GSC differentiation. (D) In contrast, *smo* ^D16^ mutant ECs are often associated with differentiating cysts. (E) *smo* ^3^ germline clones (GLCs) dissected 21 d after clone induction to show that removal of *smo* from the germ line does not affect GSC development. (F) Real-time quantitative PCR analysis of +; UAS-*smo* RNAi/SM6∧TM6B (control) and *ptc*-Gal4, UAS-*GFP*/+; UAS-*smo* RNAi/*tub*-Gal80^ts^ ovaries kept at 31°C for 7 d to show that *en* regulates positively *dpp* and *gbb* expression in niche cells. Triangles indicate statistically significant differences (Student's *t* test, *p*<0.0005). (G) *ptc*-Gal4, UAS-*GFP*/+; UAS-*dpp* RNAi/*tub*-Gal80^ts^ germarium kept at 31°C for 14 d displaying a differentiating cyst adjacent to the CpC cluster (see also Figure S5). Somatic clones were induced with the *bab1*-Gal4 driver and were dissected 3 d after eclosion. Asterisks, GSCs; white open arrowheads, wild-type TFCs; yellow open arrowheads, wild-type CpCs; red open arrowheads, wild-type ECs; yellow arrowheads, *smo* mutant CpCs; red arrowheads (all panels) and red dashed lines (D′), *smo* mutant ECs; yellow dashed lines, CpC cluster; white dashed lines, differentiating cysts within the niche. Scale bars: 10 µm.

Hh signalling is transduced intracellularly by Hh ligand binding the Patched (Ptc) receptor in receiving cells, allowing the phosphorylation and activation of Smoothened (Smo), a G-protein-coupled receptor normally inhibited by Ptc [29]. In the germarium, Hh ligand produced in the CpCs might act on GSCs directly, indirectly via ECs, or a combination of the two. To distinguish between these possibilities, we studied the expression pattern of *ptc*, itself a target gene of Hh signalling, as a readout of pathway activation. Analysis of a reporter of *ptc* expression (*ptc*-lacZ) showed expression in ECs but not in CpCs or TFCs (Figure 4A). To corroborate that activation of the *ptc* reporter responded to the canonical Hh pathway, we removed *smo* from ECs to abrogate the Hh response and found that *ptc*-lacZ expression was largely eliminated (100% of cases, *n* = 43; Figure 4B). These results indicate that the Hh pathway is active only in ECs and not in CpCs or TFCs. In fact, the generation of *smo* ^−^ CpC clones showed no effect on GSC loss by differentiation (100% of cases, *n* = 25; Figure 4C), whereas the removal of *smo* function from larval/pupal or adult ECs induced GSC differentiation, as visualised by the appearance of branched fusomes within the mosaic niches (69.56% of cases, *n* = 23; Figure 4D). Finally, the generation of mutant *smo* germline clones using two different null alleles did not result in any visible phenotypes 7, 14, or 21 d after clone induction (100% of cases, *n*>39 for each genotype and time point; Figure 4E). From these observations, we conclude that Hh produced and secreted by CpCs activates Smo in ECs to elicit a response that is responsible for GSC maintenance.

In an attempt to identify the nature of this response, we measured the mRNA levels of the essential stem cell factors *dpp* and *gbb* in Hh-depleted germaria. Real-time quantitative PCR analysis of *en* ^ts^ germaria showed that the levels of *dpp*, *gbb*, and *hh* mRNAs were reduced by more than 60% when compared to control samples (Figure S4). Because *en* is not expressed in ECs, and since *dpp* and *gbb* are transcribed in CpCs and ECs [17],[18],[22], our data indicate that *en* could regulate *dpp* and *gbb* transcription in ECs via Hh signalling. However, *dpp* has also been shown to be a target of En [30]. To test the possibility that *en* is regulating *dpp* and *gbb* transcription via *hh*, we analysed the amounts of *dpp* and *gbb* mRNAs in ovaries in which the Hh pathway was blocked specifically in ECs for 7 d (*ptc*-Gal4; UAS-*smo* RNAi/*tub*-Gal80^ts^ ovaries). In this experimental condition, the levels of *dpp* and *gbb* mRNAs are diminished by half (Figure 4F). Furthermore, these germaria also show a significant decrease in the number of GSCs per niche (Figure S5; control, 2.7±0.5 GSCs/germarium, *n* = 40; experimental, 1.4±0.6 GSCs/germarium, *n* = 44). Finally, in order to demonstrate that the expression of *dpp* in ECs is essential for GSC maintenance, we analysed niches in which *dpp* levels were diminished specifically in ECs for 14 d (*ptc*-Gal4; UAS-*dpp* RNAi/*tub*-Gal80^ts^ ovaries). We found a strong reduction in the number of GSCs due to their precocious differentiation (Figures 4G and S5; control, 2.5±0.8 GSCs/germarium, *n* = 28; experimental, 1.4±0.6 GSCs/germarium, *n* = 36). Considering that these BMP molecules are essential for GSC survival [22],[23] and that *dpp* is a target gene of the Hh pathway [31], our results support a model in which female GSC self-renewal requires the *en*-dependent production of Hh in CpCs. Upon secretion by CpCs, Hh juxtacrine signal is transmitted to the adjacent ECs, which in turn control Dpp and Gbb production to sustain GSC maintenance. The fact that the removal of *hh* from CpCs or *smo* from ECs induces a decrease in phospho-Mad levels in the germline, a direct reporter of Dpp signalling, supports this hypothesis (Figure S6). Thus, in addition to the proposed role for CpCs in ovarian niche signalling [32], ECs emerge as important regulators of niche signalling, as they not only are responsible for controlling the Jak/Stat and the EGFR pathways [12],[19] but also exert a key role in the regulation of Hh signalling.

### CpCs Respond to Impaired Hh Signalling within the Niche by Projecting Hh-Coated Cytonemes

**Figure 5:**
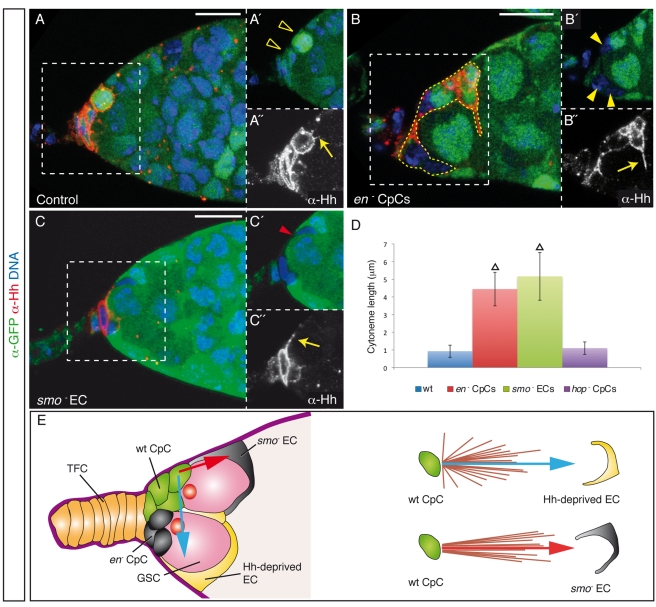
Cytoneme-mediated delivery of Hh in the niche. (A) Control germarium showing the typical short cytonemes found in CpCs of wild-type niches. (B) Mosaic germarium containing several *en* ^E^ mutant CpCs and displaying a Hh-coated long cytoneme that originates from a wild-type CpC. (C) A Hh-rich long cytoneme projecting from a wild-type CpC towards a *smo* ^D16^ mutant EC. (D) Bar chart representing the mean cytoneme length (± s.d.) in control germaria with wild-type (wt) niche cells, and in mosaic germaria containing ≥3 *en* mutant CpCs, *smo* mutant ECs, or *hopscotch* mutant CpCs (see also Figure S7 and Tables S3 and S4). Somatic clones were induced with the *bab1*-Gal4 driver and were dissected 3 d after eclosion. Triangles indicate statistically significant differences (Student's *t* test, *p*<0.0005). (E) In order to determine whether the long cytonemes found in the above mosaic germaria projected randomly within the niche, we plotted the position of these cytonemes with respect to two arbitrary axes defined as follows. In each cytoneme-containing germarium, we have drawn a straight arrow that originates in the cytoneme-growing CpC and that is oriented towards either the EC in contact with *en* mutant CpCs (*n* = 18; blue arrow) or the *smo* mutant EC (*n* = 11; red arrow). Our analysis shows that cytonemes grow directionally in the direction of the ECs in contact with *en* mutant CpCs (“Hh-deprived ECs”) or towards the *smo* mutant ECs, strongly suggesting that these long filopodia sense, and project to, the signalling-deficient region of the niche. Yellow open arrowheads, wild-type CpCs; yellow arrowheads, *en* mutant CpCs; red arrowhead, *smo* mutant EC; yellow arrows, Hh-coated cytonemes; yellow dashed line, CpC cluster. Scale bars: 10 µm.

Morphogens exert their effects over long distances, which, in the case of Hh, can be as long as 300 µm in the vertebrate limb bud [33]. In contrast, in the *Drosophila* ovarian niche, the Hh-receiving cells adjoin the Hh-producing cells, as ECs directly contact the CpC rosette, which limits the spread of this ligand. To investigate the mechanism by which Hh is transported within the ovarian niche, we analysed in detail the distribution of Hh in the CpCs. The Hh protein is strongly localised to the cell membrane, and in 30.1% of germaria analysed (*n* = 149; Figure 5A), it decorated short cellular projections 0.53 to 1.11 µm in length (0.93 µm on average) and 0.1 to 0.3 µm in diameter that formed at the CpC–EC boundaries. These narrow, filiform structures were reminiscent of the thin filopodial membranes, called cytonemes, that were initially described in the wing disc. Cytonemes are actin-rich cytoplasmic extensions thought to mediate specific morphogen signalling and to prevent inadequate diffusion of ligands [34]–[36].

In order to test the biological significance of these structures, we analysed two different experimental scenarios. First, we investigated whether these processes would respond to challenging physiological conditions such as deficient Hh niche signalling. To this end, we analysed the distribution of Hh-coated cytonemes in mosaic germaria harbouring *en* mutant CpCs and found that in 54% of these germaria one or two of the remaining wild-type CpCs displayed thin, Hh-labelled filopodia significantly longer than those of the controls (average size 3.1 µm, *n* = 50; Figure 5B and 5D). We then blocked the ability of adult ECs to respond to Hh signalling by generating *smo* ^−^ ECs, and we looked for long cytonemes in these mosaic niches. We found that the absence of Hh pathway transduction in ECs provoked a response from signalling CpCs in the form of long, Hh-coated cellular extensions detected in 50% of the cases analysed (average size 3.3 µm, *n* = 28; Figure 5C). To discard the possibility that the presence of differentiated cysts within the niche, such as those generated after removing *smo* from ECs, induces long, Hh-positive cytonemes, we generated CpCs mutant for the Jak/Stat kinase *hopscotch*, which also causes GSC differentiation [17],[18], and measured cytoneme lengths. In this condition, the cellular processes were not significantly different from those of wild-type controls (average size 1.1 µm, *n* = 20; Figure 5D). Altogether, these results clearly show that the GSC niche can react specifically to decreased Hh levels and/or to impaired Hh signalling by increasing the range of ligand spreading. Moreover, the extended cytonemes found in *en* ^−^ or *smo* ^−^ mosaic germaria projected towards the signalling-deficient area of the niche (Figure 5E), demonstrating that niche support cells sense, and respond directionally to, spatial signalling cues. Finally, to determine whether these cytonemes are specialised structures developed to mediate niche signalling, we studied the distribution of the adherens junction components *D*E-Cadherin and Armadillo in cytonemes. These proteins labelled the periphery of wild-type CpCs, delineating their round, regular shape, but were absent from their short cytonemes. Similarly, in mosaic *en* ^−^ or *smo* ^−^ niches, long cytonemes did not contain *D*E-Cadherin or Armadillo, which suggests that cytonemes are Hh-coated filopodia grown specifically to deliver a stem cell survival factor rather than a reflection of mere changes in cell shape (Figure S7).

### The Hh-Coated Cytonemes Found in Wild-Type CpCs Are Required for GSC Maintenance

**Figure 6:**
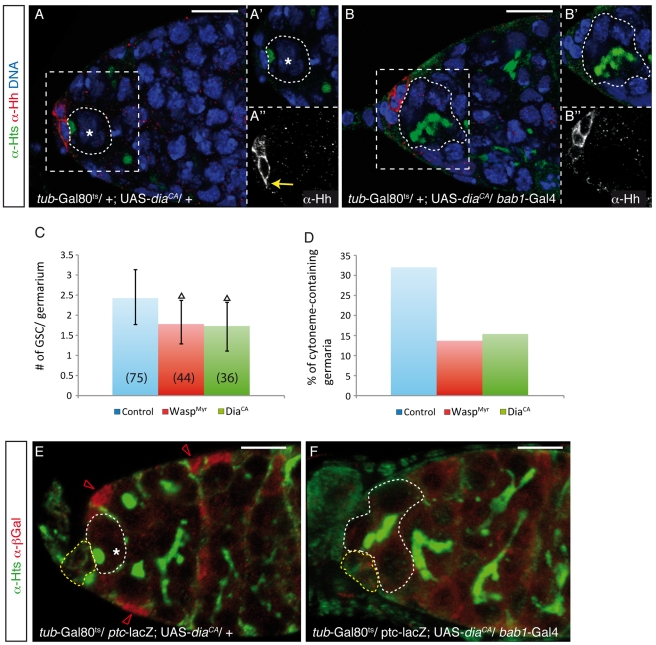
Hh-decorated cytonemes are required for GSC maintenance. (A and F) Germaria from females grown at 18°C and transferred to 31°C for 5 d upon eclosion. (A and B) Ovaries stained with anti-Hh (red) to mark cytonemes and anti-Hts (green) to label the spectrosomes and fusomes of germline cells. (A) Control germarium from a *tub*-Gal80^ts^/+; UAS-*dia* ^CA^/+ female displaying a short cytoneme (yellow arrow). (B) Experimental germarium from a *tub*-Gal80^ts^/+; *bab1*-Gal4/UAS-*dia* ^CA^ female. In this condition, the percentage of germaria showing short cytonemes and the number of GSCs per germarium are significantly lower than in controls. (C and D) Bar charts representing the mean number of GSCs (± s.d.) per germarium (C) or the percentage of germaria showing short cytonemes (D) in control *tub*-Gal80^ts^/+; UAS-*wasp* ^Myr^ (or UAS-*dia* ^CA^)/+ and in experimental *tub*-Gal80^ts^/+; *bab1*-Gal4/UAS-*wasp* ^Myr^ (Wasp^Myr^) and *tub*-Gal80^ts^/+; *bab1*-Gal4/UAS-*dia* ^CA^ (Dia^CA^) germaria. The number of germaria analysed for each experiment (*n*) is shown. Black triangles indicate a statistically significant difference between the given experimental condition and the control (Student's *t* test, *p*<0.01). The percentages of experimental germaria containing cytonemes were significantly different from controls with a probability of 95% (Chi-square test). (E and F) The overexpression of Dia^CA^ largely abrogates the activation of the Hh pathway in ECs, as shown by the absence of *ptc*-lacZ expression—a target of the pathway—in experimental germaria. Asteriks, GSCs; red open arrowheads, ECs showing *ptc*-lacZ expression; yellow dashed lines, CpC clusters; white dashed lines, GSCs in (A) and (E) and differentiating cysts within the niche in (B) and (F). Scale bars: 10 µm.

Next, we wished to study cytoneme functionality by affecting their formation. Because cytonemes are rich in actin filaments [34], we reasoned that disturbing actin polymerisation in adult CpCs could have an effect specifically on cytoneme production and/or kinetics. Thus, we utilised the *bab1*-Gal4 driver to express modified versions of two known regulators of actin polymerisation in TFCs and CpCs of adult ovaries. We induced the expression of either a constitutively activated form of the *Drosophila* Formin homologue Diaphanus (Dia), Dia^CA^ [37], or a myristoylated form of the Arp2/3-complex regulator Wasp, Wasp^Myr^ [38]. While interfering with actin polymerisation may affect other cellular processes rather than cytoneme formation, we performed several controls to make sure that the observed results where as specific as possible. First, we measured the mean value of fluorescence intensity per area unit in control (*tub*-Gal80^ts^/+; UAS-*dia* ^CA^/+ or *tub*-Gal80^ts^/+; UAS-*wasp* ^Myr^/+) or experimental ovaries (*tub*-Gal80^ts^/+; UAS-*dia* ^CA^/*bab1*-Gal4 or *tub*-Gal80^ts^/+; UAS-*wasp* ^Myr^/*bab1*-Gal4) kept at 31°C for 5 d upon eclosion to confirm that overexpression of UAS-*wasp* ^Myr^ or UAS-*dia* ^CA^ in adult germaria affected significantly neither the overall amounts of Hh protein in the niche cells nor the expression of CpC markers such as *bab1* or Lamin C (Figure 6 and data not shown). Second, we manipulated only post-mitotic cells to prevent unwanted effects during mitosis, as we induced ectopic gene expression in adult CpCs. Third, we utilised an experimental setting that did not affect visibly niche morphology or CpC viability. In this scenario, we found that ectopic expression of Wasp^Myr^ or Dia^CA^ for 5 d in niche cells halved the number of germaria growing short cytonemes (from over 30% in controls to 13.6% and 15.4%, respectively, *n*>36 for each genotype; Figure 6C). Interestingly, this condition also produced a significant decrease in the number of GSCs per niche (from 2.45±0.7 in controls to 1.8±0.55 and 1.7±0.6, respectively, *n*>36 for each genotype; Figure 6D). Since we did not observe apoptosis above control levels in germline cells (data not shown) and because we could detect differentiating cysts in these experimental niches (Figure 6B), the formation of short Hh-decorated filopodia in CpCs is an essential step to prevent GSC differentiation. We next tested whether diminishing the number of cytonemes per CpC would affect Hh signalling. To this end, we overexpressed Dia^CA^ in adult TFCs and CpCs utilising the *bab1*-Gal4 driver and monitored the activation of the Hh-signalling reporter *ptc*-lacZ. We found that, in contrast to controls, experimental females grown for 5 d at 31°C largely failed to activate the *ptc*-lacZ reporter in the germarium (Figure 6E and 6F). These results, together with our previous finding that Hh is produced in CpCs and received in ECs, strongly suggest that the Hh-coated cytonemes regulate Hh signalling in the germarium by facilitating Hh delivery to the target ECs to ensure that a normal pool of stem cells is maintained.

## Discussion

**Figure 7:**
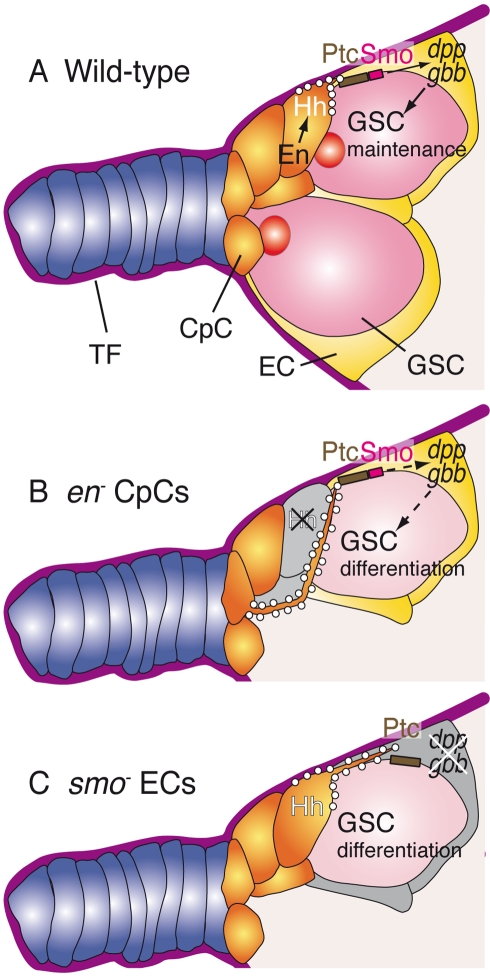
A model for the response of the GSC niche to low levels of Hh signalling. (A) In wild-type niches En regulates Hh production in CpCs, which is then delivered to the neighbouring ECs via short, thin cytoplasmic extensions. Upon binding to the Ptc receptor, thus releasing Smo from inhibition, the Hh signal is transduced in ECs to regulate Dpp and Gbb production, which in turn act as survival factors to maintain the GSC population within the niche. (B) Mosaic niche containing *en* mutant CpCs unable to produce the Hh ligand. As a result, the transcription of the *dpp* and *gbb* genes in the adjacent ECs is compromised, resulting in GSC loss. Wild-type CpCs respond to this deficiency in Hh levels by directing Hh-rich long cytonemes towards the Hh-deprived ECs. (C) Blocking *smo* function prevents target gene activation and causes GSC depletion. Wild-type CpCs react to this deficient readout of Hh signalling by projecting Hh-coated long cytonemes towards the *smo* mutant ECs.

Niches are dynamic systems often containing stromal cells that provide physical support and survival factors to nurture a population of stem cells. The data presented here demonstrate that the heterotypic association of support cells is crucial for niche function. In the case of the *Drosophila* ovarian niche, it has been previously described that the Jak/Stat pathway regulates the expression of *dpp* in CpCs [17],[18]. Our results show that the maintenance of a stable population of GSCs relies also on the coordinated action of the CpCs and the ECs, which allows the production and release of the GSC survival ligand Hh in the CpCs and its reception in the ECs. As a consequence of the transduction of the Hh pathway, ECs produce the stem cell factors Dpp and Gbb (see model in Figure 7). The recent finding of a similar partnership between mesenchymal and haematopoietic stem cells that operates in the bone marrow niche [39] indicates that such collective regulatory interactions within support cells may be a common feature of cellular niches.

The study of the mechanisms behind Hh signalling in the *Drosophila* ovary has allowed the identification of Hh-coated cytonemes in a cellular stem cell niche, emphasising the idea that cytonemes mediate spreading of the activating signal from the producing cells. Recently, it has been reported that the Hh protein localises to long, basal cellular extensions in the wing disk [40]. In addition, filopodial extensions in the wing, eye, and tracheal system of *Drosophila* have been shown to segregate signalling receptors on their surface, thus restricting the activation of signalling pathways in receiving cells [36]. Hence, cytonemes, as conduits for signalling proteins, may be extended by receiving cells—and so are involved in uptake—or may be extended by producing cells—and so are involved in delivery and release.

Interfering with actin polymerisation in adult niches leads to a significant reduction in the number of CpCs growing Hh cytonemes, concomitant with precocious stem cell differentiation, demonstrating that these actin-rich structures are required to prevent stem cell loss and thus are functionally relevant. Importantly, because we disturbed actin dynamics in post-mitotic CpCs that still produce wild-type levels of Hh protein and express CpC markers (but fail to activate the Hh pathway in ECs), the observed effects on stem cell maintenance are most likely specific to Hh delivery from CpCs to their target ECs via short cytonemes. This interpretation is further reinforced by the observation that CpCs can sense decreased Hh levels and/or a dysfunction in the transduction of the Hh pathway in the niche and respond to it by growing Hh-rich membrane bridges up to 6-fold longer than in controls. In this regard, it is interesting to note that the two lipid modifications found in mature Hh act as membrane anchors and give secreted Hh a high affinity for membranes and signalling capacities [41],[42]. In fact, it has been recently described that a lipid-unmodified form of Hh unable to signal does not decorate filopodia-like structures in the wing imaginal disc epithelium, confirming the link between Hh transport along cytonemes and Hh signalling [40]. Thus, cytonemes may ensure specific targeting of the Hh ligand to the receiving germline cells in a context of intense signalling between niche cells and the GSCs. Interestingly, in both *en* ^−^ and *smo* ^−^ mosaic niches, the long processes projected towards the signalling-deficient area of the niche, which showed that competent CpCs sense the strength of Hh signalling activity in the microenvironment. While the nature of the signal perceived by the CpCs or the receptor(s) involved in the process are unknown, we postulate that Hh-decorated filopodial extensions represent the cellular synapsis required for signal transmission that is established between the Hh-producing cells (the CpCs) and the Hh-receiving cells (the ECs). In this scenario—and because Ptc, the Hh receptor, is a target of the pathway—the membranes of mutant ECs, in which the transduction of the pathway is compromised, contain lower Ptc levels. Thus, longer and perhaps more stable projections ought to be produced to allow proper signalling. In addition, the larger the number of *en* mutant cells (and hence the stronger the deficit in Hh ligand concentration or target gene regulation), the longer the cellular projections decorated with Hh (Tables S3 and S4), which indicates that the niche response is graded depending on the degree of signalling shortage.

Do the longer cytonemes found in mosaic germaria represent structures created de novo, or do they simply reflect a pre-existing meshwork of thin intercellular bridges that can regulate the amount of Hh protein in transit across them? Because we utilised an anti-Hh antibody to detect the cytonemes and all of our attempts to identify other markers for these structures have failed, we cannot presently discriminate between these two possibilities. In any case, since we did not detect increased Hh levels in wild-type CpCs that contained cytonemes relative to those that did not, it is clear that long filopodia do not arise solely by augmenting Hh production in the CpCs. Rather, if long cytonemes are not synthesised in response to a Hh signalling shortage and if they already existed in the niche, they ought to restrict Hh spreading independently of significant Hh production. Furthermore, because the strength of Hh signalling in the niche determines the distance of Hh spreading, either cytoneme growth or Hh transport (or both) are regulated by the ability of the CpCs to sense the Hh signalling output.

Our demonstration that a challenged GSC niche can respond to insufficient signalling by the cytoneme-mediated delivery of the stem cell survival factor Hh over long distances has wider implications. Niche cells have been shown to send cellular processes to their supporting stem cells in several other scenarios: the *Drosophila* ECs of the ovary and the lymph gland, the ovarian niche of earwigs, and the germline mitotic region in the hermaphrodite *Caenorhabditis elegans* [5],[6],[14],[43],[44]. Similarly, wing and eye disc cells project cytonemes to the signalling centre of the disc [34],[36]. However, definitive proof that the thin filopodia described in the lymph gland, the earwig ovary, or imaginal discs deliver signals from the producing to the effector cells is lacking. Our findings strongly suggest that cytonemes have a role in transmitting niche signals over distance, a feature that may underlie the characteristic response of more complex stem cell niches to challenging physiological conditions. Careful analysis of the architecture of sophisticated niches, such as the bone marrow trabecular zone for mouse haematopoietic stem cells, will be needed to further test this hypothesis and to determine whether it represents a conserved mechanism for stem cell niche signalling.

## Materials and Methods

### Fly Stocks

Flies were grown at 25°C on standard medium for *Drosophila*. The following genetically null alleles were used: *en* ^E^, *en* ^54^ [45], *hh* ^21^, *hh* ^AC^ [46], *disp* ^SH21^ [28], *smo* ^D16^ [47], and *smo* ^3^ [48]. *ptc* ^AT96^ is a *LacZ* enhancer trap inserted in the gene [49]. *en* ^spt^ [50] is a temperature-sensitive allele. To express UASt-*DsRed* and UASt-*flp* we used the *bab1*-Gal4 line [51]. The expression of UAS transgenes in ECs was done utilising the *ptc*-Gal4 driver. In order to generate experimental *en* ^spt^/*en* ^E^ adult females, flies were shifted from 25°C to 28°C for 7 or 14 d upon eclosion.

To obtain adult females overexpressing Wasp^Myr^ [38] or Dia^CA^ [37], *w*; *tub*-Gal80^ts^/CyO; *bab1*-Gal4/TM2 flies were crossed to *w*; UAS-*wasp* ^Myr^ or *w*; UAS-*dia* ^CA^, respectively. To overexpress *dpp* RNAi (VDRC) or *smo* RNAi (Bloomington Stock Center) in ECs, *w*; *ptc*-Gal4, UAS-*GFP*; *tub*-Gal80^ts^/SM6∧TM6B flies were crossed to *w*; UAS-*dpp* RNAi, *w*; UAS-*smo* RNAi. The offspring were grown at 18°C, and upon eclosion adult F1 flies were shifted to 31°C for 5, 7, or 14 d.

### Immunohistochemistry and Image Analysis

Ovaries were dissected at room temperature in PBS containing 0.1% Tween-20 (PBT), fixed for 20 min with 4% PFA, blocked with PBT+10% BSA for 1 h, and washed in PBT before they were incubated for 15 h with primary antibodies diluted in PBT supplemented with 1% BSA. Primary antibodies were washed three times in PBT containing 1% BSA. Secondary antibodies were diluted in PBT containing 0.1% BSA. Primary antibodies were used at the following concentrations: mouse anti-Hts (1B1) (Developmental Studies Hybridoma Bank [DSHB], University of Iowa), 1∶50; rabbit anti-Vasa (a gift from R. Lehmann), 1∶1,000; mouse anti-En (4D9) (DSHB), 1∶50; rabbit anti-α-Spectrin (a gift from R. Dubreuil), 1∶400; rabbit anti-GFP (Molecular Probes), 1∶500; mouse anti-GFP (Molecular Probes), 1∶50; mouse anti–Lamin C (LC28.26) (DSHB), 1∶50; rabbit anti-Hh (a gift from S. Eaton [52]), 1∶500; rabbit anti-phospho-Mad 1/5/8 (a gift from E. Laufer), 1∶5,000; rabbit anti-β-galactosidase (Cappel), 1∶1,000; rabbit anti-cleaved Caspase 3 (BioLabs), 1∶50; and mouse anti-Orb (6H4+4H8) (DSHB), 1∶50. Secondary antibodies (Cy2- and Cy3-conjugated, Jackson ImmunoResearch) were used at 1∶100. DNA staining was performed using the DNA dye Hoechst (Sigma) at 1∶1,000. Images were captured with a Leica SPE confocal microscope and processed using ImageJ, Adobe Photoshop, and Adobe Illustrator.

Fluorescence intensity units and cytoneme length were measured using the Leica LAS-AF software. Images were captured with a Leica SPE confocal microscope and processed using ImageJ, Adobe Photoshop, and Adobe Illustrator.

### Generation of Somatic and Germline Mitotic Clones

To generate mitotic clones we induced the Flipase enzyme using either a heat shock promoter or the *bab1*-Gal4 driver to activate expression of a UAS-*flp* construct. *en* and *smo* mutant germline clones were induced by giving 3-d-old females two 1-h-long heat shocks at 37°C spaced by 10 h at 25°C. *hh*, *disp*, *en*, and *smo* mutant somatic clones were induced expressing UAS-*flp* with the *bab1*-Gal4 driver. Ovaries were processed 3 d (for somatic clones) or 7, 14, or 21 d (for germline clones) after treatment. To eliminate *smo* function in adult females, 3-d-old HS-*flp* ^1112^/+; *smo* ^D16^ FRT40A/*ubi-nls*:GFP FRT40A flies were subjected to three 1-h-long heat shocks at 37°C separated by 6-h periods at 25°C. The following chromosomes were used: HS-*flp* ^1112^, FRT42D *en* ^54^, FRT42D *en* ^E^, FRT42D *ubi-nls*:GFP, *smo* ^D16^ FRT40A, *ubi-nls*:GFP FRT40A, FRT42D *ubi-nls*:GFP, *hh* ^AC^ FRT82B, *hh* ^21^ FRT82B, *disp* ^SH21^ FRT82B, *bab1*-Gal4 FRT82B *ubi-nls*:GFP, UASt-*flp*, *smo* ^3^ FRT40A *ptc* ^AT96^, and *bab1*-Gal4 UASt-*flp*.

### Quantification of *hh*, *dpp*, and *gbb* mRNA Levels

The relative amounts of *hh*, *dpp*, and *gbb* mRNAs were determined by real-time quantitative PCR using the comparative cycle threshold (*C*_T_) method [53], Fam-dye-labelled TaqMan MGB probes (Applied Biosystems), and an ABI-PRISM 7700 Sequence Detection System. RNA polymerase II (*RpII140*) was used to normalise mRNA levels. *hh*, *dpp*, or *gbb* mRNA relative amount was calculated from the determination of the difference between the *C*_T_ of the given gene and that of *RpII140*. *C*_T_ values used were the result of three different replicas from three independent experiments. Primers and TaqMan probes for the different cDNAs were obtained from the Assays-by-Design Service (Applied Biosystems) with the following sequences (5′–3′): *RpII140*, forward, ACTGAAATCATGATGTACGACAACGA, reverse, TGAGAGATCTCCTCGGCATTCT, probe, TCCTCGTACAGTTCTTCC; *hh*, forward, GCAGGCGCCACATCTACT, reverse, GCACGTGGGAACTGATCGA, probe, CCGTCAAGTCAGATTCG; *dpp*, forward, GCCAACACAGTGCGAAGTTTTA, reverse, TGGTGCGGAAATCGATCGT, probe, CACACAAAGATAGTAAAATC; *gbb*, forward, CGCTGTCCTCGGTGAACA, reverse, CGGTCACGTTGAGCTCCAA, probe, CCAGCCCACGTAGTCC.

cDNA was synthesised from ∼100–200 ovary pairs of the following characteristics: *en* ^spt^/CyO (control) and *en* ^spt^ */en* ^E^ (experimental) females were shifted from 25°C to 28°C for 7 d after eclosion prior to dissection. +; UAS-*smo* RNAi/SM6∧TM6B (control) and *ptc*-Gal4, UAS-*GFP*/+; UAS-*smo* RNAi/*tub*-Gal80^ts^ (experimental) females were shifted from 25°C to 31°C for 7 d after eclosion prior to dissection.

### Statistical Analysis

A Student's *t* test was used to determine whether the following were significantly different between control and experimental samples: (i) the mean number of GSCs and differentiated cysts per germarium, (ii) the relative levels of *hh*, *dpp*, and *gbb* expression, and (iii) the length of cytonemes. To analyse whether the observed differences in the percentages of cytoneme-containing germaria between control ovaries and ovaries overexpressing Wasp^Myr^ or Dia^CA^ were significant, we applied the Chi-square test. Differences were considered significant when the *p*-values were less than 0.01.

## Supporting Information

Figure S1 **Loss of** ***en*** **function in CpCs induces GSC differentiation.** This supplemental figure is related to Figure 2. (A–C) FRT42D *en* ^E^/FRT42D *ubi*-*nls*:GFP; *bab1*-Gal4 UASt-*flp* germaria. (A and A′) Control germarium stained with anti-Orb (red), anti-GFP (green), and Hoechst (white) to show the progressive accumulation of Orb protein in the oocyte of differentiating cysts. (B and B′) Experimental germarium containing *en* mutant CpCs. The germline cyst adjacent to the CpCs already shows Orb protein accumulated in a single cell, a characteristic of mature 16-cell cysts. (C and C′) Germarium stained with the apoptotic marker anti-Caspase 3 to show that the loss of *en* in the CpCs does not induce GSC apoptosis. Rather, these cells enter differentiation. Asterisks, GSCs; yellow open arrowheads, wild-type CpCs; yellow dashed lines, *en* mutant CpCs; white dashed lines, differentiating germline cysts. Scale bars: 10 µm.
(PDF)

Figure S2 **The loss of** ***en*** **does not affect CpC fate.** This supplemental figure is related to Figure 2. (A–A″) FRT42D *en* ^E^/FRT42D *ubi*-*nls*:GFP; *bab1*-Gal4 UASt-*flp* germarium stained with anti-Lamin C to visualise TFCs and CpCs (red), anti-GFP to mark mutant cells (green), and Hoechst (for DNA; blue). (B–B″) *w*; UASt-*DsRed*; FRT42D *en* ^E^/FRT42D *ubi*-*nls*:GFP; *bab1*-Gal4 UASt-*flp* germarium stained with anti-GFP to mark mutant cells (green) and Hoechst (for DNA; blue). The autofluorescence of the DsRed protein was observed directly. The expression of the *bab1* gene and of Lamin C protein are not altered in *en* mutant CpCs (yellow arrowheads). Scale bars: 10 µm.
(PDF)

Figure S3 **Hh-positive cells at the base of the terminal filament express the CpC marker Lamin C.** This supplemental figure is related to Figure 3. (A–A″) Wild-type germarium stained with anti-Lamin C (red), anti-Hh (green), and Hoechst (blue). Yellow open arrowheads, wild-type CpCs. Scale bar: 10 µm.
(PDF)

Figure S4 ***hh*** **,** ***dpp*** **, and** ***gbb*** **mRNA levels are decreased in** ***en*** **^spt^ mutant ovaries.** This supplemental figure is related to Figure 4. Real-time quantitative PCR analysis of *en* ^spt^/CyO (control) and *en* ^spt^/*en* ^E^ ovaries kept at 28°C for 7 d to show that *en* regulates positively *hh*, *dpp*, and *gbb* expression in niche cells. In wild-type germaria, *en* is expressed in TFCs and CpCs. Triangles indicate statistically significant differences (Student's *t* test, *p*<0.0005).
(PDF)

Figure S5 **The reduction of** ***dpp*** **or** ***smo*** **mRNA levels in ECs induces GSC loss.** This supplemental figure is related to Figure 4. Overexpression of *dpp* or *smo* RNAi in ECs utilising the *ptc*-Gal4 driver reduces the number of GSCs per germarium. Control females (+; UAS-*dpp* RNAi/SM6∧TM6B and +; UAS-*smo* RNAi/SM6∧TM6B) and experimental females (*ptc*-Gal4, UAS-*GFP*/+; UAS-*dpp* RNAi/*tub*-Gal80^ts^ or *ptc*-Gal4, UAS-*GFP*/+; UAS-*smo* RNAi/*tub*-Gal80^ts^) were transferred from 18°C to 31°C for 7 or 14 d after eclosion. The total amount of GSCs per germarium was determined by counting the number of spectrosome-containing germline cells in contact with CpCs. Triangles indicate statistically significant differences (Student's *t* test, *p*<0.0001). The sample size (number of germaria analysed) is shown for each genotypic class.
(PDF)

Figure S6 **The activity of the** ***dpp*** **pathway in the germline depends on the expression of** ***hh*** **in CpCs or that of** ***smo*** **in ECs.** This supplemental figure is related to Figure 4. (A and B) UASt-*flp*/+; FRT82B *hh* ^AC^/*bab1*-Gal4 FRT82B *ubi*-*nls*:GFP and (C) *smo* ^D16^ FRT40A/*ubi*-*nls*:GFP FRT40A; *bab1*-Gal4 UASt-*flp*/+ germaria stained with anti-phospho-Mad (red), anti-GFP (green), and Hoechst (blue) to show that the activation of the *dpp* pathway—and thus the expression of phospho-Mad—in the GSCs and cystoblasts depends on the production of Hh in the CpCs and the activation of its pathway via Smo in the ECs. (A and A′) Control germarium showing the accumulation of phospho-Mad in GSCs (white asterisks) and, to a lesser extent, in cystoblasts (yellow asterisks). (B and C) Experimental germaria containing *hh* mutant CpCs (B and B′) or *smo* mutant ECs (C and C′). Germline cells adjacent to mutant cells do not express detectable levels of phospho-Mad. White asterisks, GSCs; yellow asterisk, cystoblast; yellow open arrowheads, wild-type CpCs; red open arrowheads, wild-type ECs; yellow arrowheads, *hh* mutant CpCs; red arrowheads, *smo* mutant ECs. Scale bars: 10 mm.
(PDF)

Figure S7 **Projected cytonemes do not contain the adherent junction components Armadillo or** ***D*** **E-Cadherin.** This supplemental figure is related to Figure 5. (A) In wild-type niches, Hh and Armadillo co-localise at the cell periphery in CpCs. (B and C) FRT42D *en* ^E^/FRT42D *ubi*-*nls*:GFP; *bab1*-Gal4 UASt-*flp* mosaic germaria containing *en* mutant cells and stained for anti-Hh and anti-Arm (B) or anti-*D*E-Cadherin (C). In these mosaic germaria, some wild-type CpCs project long cytonemes decorated with Hh protein. However, these filopodia do not contain *D*E-Cadherin or Armadillo. Yellow open arrowheads point to CpCs. Yellow arrows demarcate Hh-containing cytonemes. Yellow open arrows indicate the absence of *D*E-Cadherin or Armadillo in these filopodia. Scale bars: 10 mm.
(PDF)

Table S1 **The number of GSCs per germarium depends on** ***en*** **activity.** This supplemental table is related to Figure 1. The percentage of germaria containing 0, 1, or 2–3 GSCs is shown for four different genotypes. Females were shifted from 25°C to 28°C for 7 d upon eclosion and prior to dissection.
(DOC)

Table S2 **The average number of GSCs in mosaic germaria depends on the number of** ***hh*** **mutant CpCs.** This supplemental table is related to Figure 5. The table shows the average number of GSCs in control and experimental germaria containing ≤2 or ≥3 *hh* mutant CpCs.
(DOC)

Table S3 **Average cytoneme length in different experimental conditions.** This supplemental table is related to Figure 5. The table shows the average length (in micrometers) of cytonemes that project from wild-type CpCs in wild-type controls and in mosaic germaria containing 1, ≤2, or ≥3 mutant cells.
(DOC)

Table S4 **Total cytoneme length per CpC in different experimental conditions.** This supplemental table is related to Figure 5. The table shows the average length (in micrometers) of all cytonemes per CpC in wild-type controls and in mosaic germaria containing 1, ≤2, or ≥3 mutant cells.
(DOC)

## Abbreviations

BMP: Bone Morphogenetic Protein
CpC: cap cell
Dia: Diaphanus
*disp*: *dispatched*
Dpp: Decapentaplegic
DSHB: Developmental Studies Hybridoma Bank
EC: escort cell
En: Engrailed
Gbb: Glass bottom boat
GSC: germline stem cell
Hh: Hedgehog
Jak/Stat: Janus kinase/Signal transducer and activator of transcription
PBT: PBS containing 0.1% Tween-20
Ptc: Patched
s.d.: standard deviation
Smo: Smoothened
TFC: terminal filament cell
