# Supplementary figures and images for: Cytoneme-Mediated Delivery of Hedgehog Regulates the Expression of Bone Morphogenetic Proteins to Maintain Germline Stem Cells in Drosophila
Source: PLoS Biol. 2012 Apr 3;10(4):e1001298. doi: 10.1371/journal.pbio.1001298 (PMC3317903; doi:10.1371/journal.pbio.1001298)

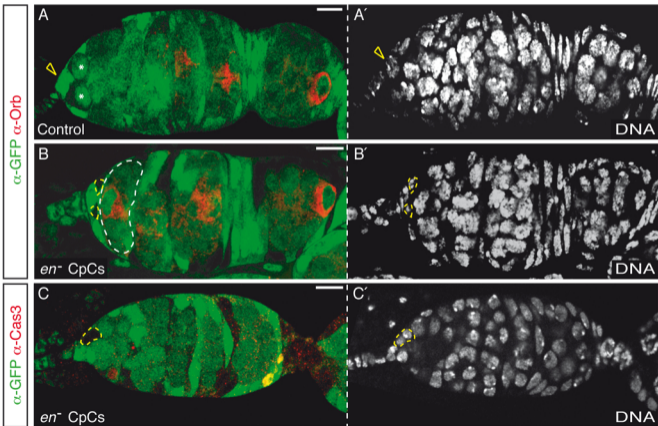

Supplement: Figure S1 — Loss of en function in CpCs induces GSC differentiation. This supplemental figure is related to Figure 2. (A–C) FRT42D en E/FRT42D ubi-nls:GFP; bab1-Gal4 UASt-flp germaria. (A and A′) Control germarium stained with anti-Orb (red), anti-GFP (green), and Hoechst (white) to show the progressive accumulation of Orb protein in the oocyte of differentiating cysts. (B and B′) Experimental germarium containing en mutant CpCs. The germline cyst adjacent to the CpCs already shows Orb protein accumulated in a single cell, a characteristic of mature 16-cell cysts. (C and C′) Germarium stained with the apoptotic marker anti-Caspase 3 to show that the loss of en in the CpCs does not induce GSC apoptosis. Rather, these cells enter differentiation. Asterisks, GSCs; yellow open arrowheads, wild-type CpCs; yellow dashed lines, en mutant CpCs; white dashed lines, differentiating germline cysts. Scale bars: 10 µm. (PDF) [file pbio.1001298.s001.pdf]

# Supplemental Figure 2

Rojas-Ríos et al.

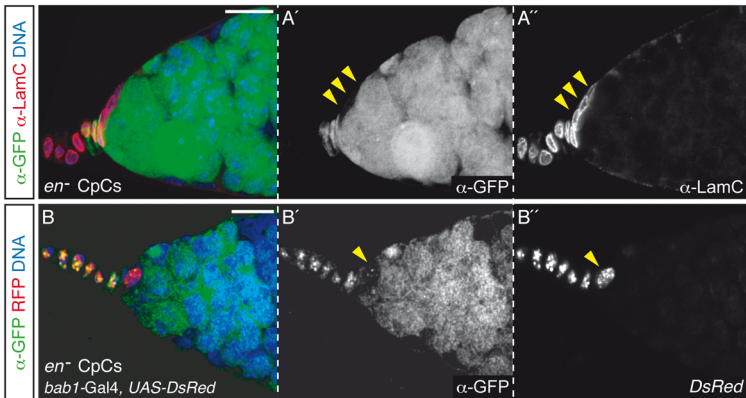

Supplement: Figure S2 — The loss of en does not affect CpC fate. This supplemental figure is related to Figure 2. (A–A″) FRT42D en E/FRT42D ubi-nls:GFP; bab1-Gal4 UASt-flp germarium stained with anti-Lamin C to visualise TFCs and CpCs (red), anti-GFP to mark mutant cells (green), and Hoechst (for DNA; blue). (B–B″) w; UASt-DsRed; FRT42D en E/FRT42D ubi-nls:GFP; bab1-Gal4 UASt-flp germarium stained with anti-GFP to mark mutant cells (green) and Hoechst (for DNA; blue). The autofluorescence of the DsRed protein was observed directly. The expression of the bab1 gene and of Lamin C protein are not altered in en mutant CpCs (yellow arrowheads). Scale bars: 10 µm. (PDF) [file pbio.1001298.s002.pdf]

# Supplemental Figure 3

Rojas-Ríos et al.

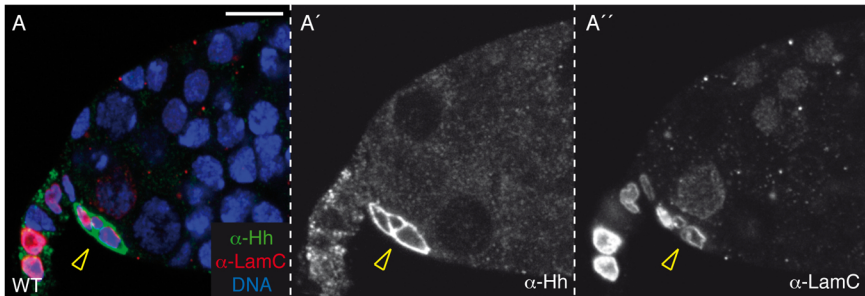

Supplement: Figure S3 — Hh-positive cells at the base of the terminal filament express the CpC marker Lamin C. This supplemental figure is related to Figure 3. (A–A″) Wild-type germarium stained with anti-Lamin C (red), anti-Hh (green), and Hoechst (blue). Yellow open arrowheads, wild-type CpCs. Scale bar: 10 µm. (PDF) [file pbio.1001298.s003.pdf]

## Supplemental Figure 4

Rojas-Ríos et al.

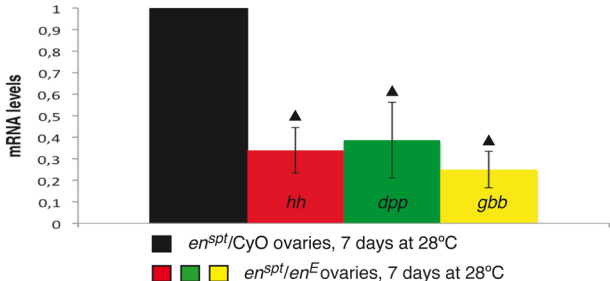

Supplement: Figure S4 — hh , dpp , and gbb mRNA levels are decreased in en spt mutant ovaries. This supplemental figure is related to Figure 4. Real-time quantitative PCR analysis of en spt/CyO (control) and en spt/en E ovaries kept at 28°C for 7 d to show that en regulates positively hh, dpp, and gbb expression in niche cells. In wild-type germaria, en is expressed in TFCs and CpCs. Triangles indicate statistically significant differences (Student's t test, p<0.0005). (PDF) [file pbio.1001298.s004.pdf]

# Supplemental Figure 5

Rojas-Ríos et al.

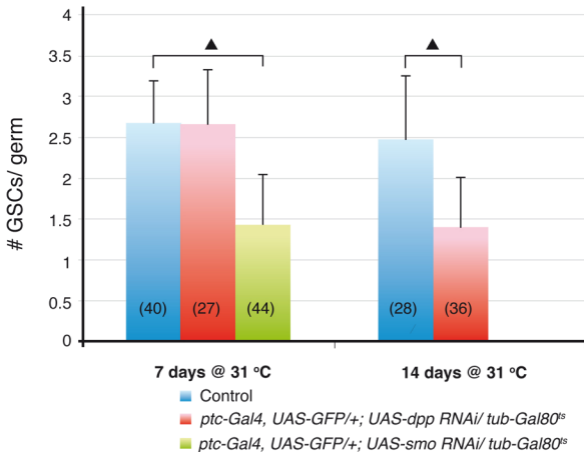

Supplement: Figure S5 — The reduction of dpp or smo mRNA levels in ECs induces GSC loss. This supplemental figure is related to Figure 4. Overexpression of dpp or smo RNAi in ECs utilising the ptc-Gal4 driver reduces the number of GSCs per germarium. Control females (+; UAS-dpp RNAi/SM6∧TM6B and +; UAS-smo RNAi/SM6∧TM6B) and experimental females (ptc-Gal4, UAS-GFP/+; UAS-dpp RNAi/tub-Gal80ts or ptc-Gal4, UAS-GFP/+; UAS-smo RNAi/tub-Gal80ts) were transferred from 18°C to 31°C for 7 or 14 d after eclosion. The total amount of GSCs per germarium was determined by counting the number of spectrosome-containing germline cells in contact with CpCs. Triangles indicate statistically significant differences (Student's t test, p<0.0001). The sample size (number of germaria analysed) is shown for each genotypic class. (PDF) [file pbio.1001298.s005.pdf]

# Supplemental Figure 6

Rojas-Ríos et al.

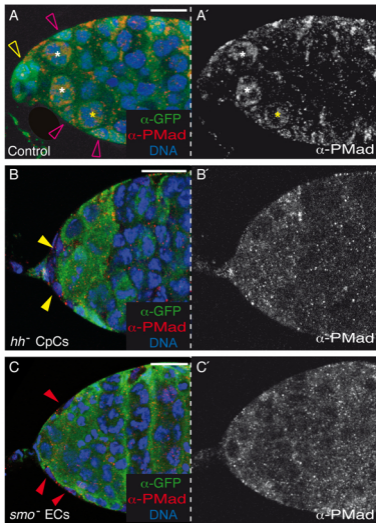

Supplement: Figure S6 — The activity of the dpp pathway in the germline depends on the expression of hh in CpCs or that of smo in ECs. This supplemental figure is related to Figure 4. (A and B) UASt-flp/+; FRT82B hh AC/bab1-Gal4 FRT82B ubi-nls:GFP and (C) smo D16 FRT40A/ubi-nls:GFP FRT40A; bab1-Gal4 UASt-flp/+ germaria stained with anti-phospho-Mad (red), anti-GFP (green), and Hoechst (blue) to show that the activation of the dpp pathway—and thus the expression of phospho-Mad—in the GSCs and cystoblasts depends on the production of Hh in the CpCs and the activation of its pathway via Smo in the ECs. (A and A′) Control germarium showing the accumulation of phospho-Mad in GSCs (white asterisks) and, to a lesser extent, in cystoblasts (yellow asterisks). (B and C) Experimental germaria containing hh mutant CpCs (B and B′) or smo mutant ECs (C and C′). Germline cells adjacent to mutant cells do not express detectable levels of phospho-Mad. White asterisks, GSCs; yellow asterisk, cystoblast; yellow open arrowheads, wild-type CpCs; red open arrowheads, wild-type ECs; yellow arrowheads, hh mutant CpCs; red arrowheads, smo mutant ECs. Scale bars: 10 mm. (PDF) [file pbio.1001298.s006.pdf]

# Supplemental Figure 7

Rojas-Ríos et al.

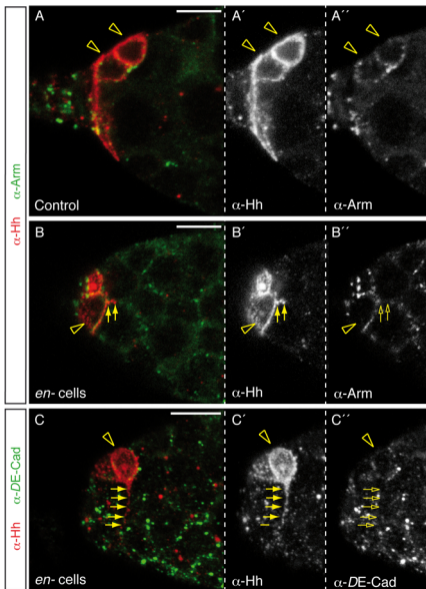

Supplement: Figure S7 — Projected cytonemes do not contain the adherent junction components Armadillo or D E-Cadherin. This supplemental figure is related to Figure 5. (A) In wild-type niches, Hh and Armadillo co-localise at the cell periphery in CpCs. (B and C) FRT42D en E/FRT42D ubi-nls:GFP; bab1-Gal4 UASt-flp mosaic germaria containing en mutant cells and stained for anti-Hh and anti-Arm (B) or anti-DE-Cadherin (C). In these mosaic germaria, some wild-type CpCs project long cytonemes decorated with Hh protein. However, these filopodia do not contain DE-Cadherin or Armadillo. Yellow open arrowheads point to CpCs. Yellow arrows demarcate Hh-containing cytonemes. Yellow open arrows indicate the absence of DE-Cadherin or Armadillo in these filopodia. Scale bars: 10 mm. (PDF) [file pbio.1001298.s007.pdf]
