# Supplementary material for: Cytoneme-Mediated Delivery of Hedgehog Regulates the Expression of Bone Morphogenetic Proteins to Maintain Germline Stem Cells in Drosophila
Source: PLoS Biol. 2012 Apr 3;10(4):e1001298. doi: 10.1371/journal.pbio.1001298 (PMC3317903; doi:10.1371/journal.pbio.1001298)
Supplement: Table S1 — The number of GSCs per germarium depends on en activity. This supplemental table is related to Figure 1. The percentage of germaria containing 0, 1, or 2–3 GSCs is shown for four different genotypes. Females were shifted from 25°C to 28°C for 7 d upon eclosion and prior to dissection. (DOC) [file pbio.1001298.s008.doc]

| **Genotype (n)** | **2-3 GSCs/ niche** | **1 GSC/ niche** | **0 GSCs/ niche** |
| --- | --- | --- | --- |
| *w^1118^* (78) | 100% | 0% | 0% |
| *en^spt^*/ CyO (62) | 83.9% | 14.5% | 1.6% |
| *en^spt^* (116) | 58% | 46.4% | 17.4% |
| *en^spt^*/ *en^E^* (35) | 45.7% | 25.7% | 28.6% |
