# Supplementary material for: Cytoneme-Mediated Delivery of Hedgehog Regulates the Expression of Bone Morphogenetic Proteins to Maintain Germline Stem Cells in Drosophila
Source: PLoS Biol. 2012 Apr 3;10(4):e1001298. doi: 10.1371/journal.pbio.1001298 (PMC3317903; doi:10.1371/journal.pbio.1001298)
Supplement: Table S2 — The average number of GSCs in mosaic germaria depends on the number of hh mutant CpCs. This supplemental table is related to Figure 5. The table shows the average number of GSCs in control and experimental germaria containing ≤2 or ≥3 hh mutant CpCs. (DOC) [file pbio.1001298.s009.doc]

| **Genotype of CpCs** | | **Mean number of GSCs ± s. d. (n)** |
| --- | --- | --- |
| Wild-type | All CpCs | 2.6 ± 0.5 (30) |
| *hh^21-^*CpCs | ≤ 2 cells | 1.86 ± 0.82 (7) |
|  | ≥ 3 cells | 0.82 ± 0.64 (18) |
| *hh^AC-^* CpCs | ≤ 2 cells | 2 ± 0.82 (4) |
|  | ≥ 3 cells | 0.8 ± 1.10 (15) |
