# Supplementary material for: Cytoneme-Mediated Delivery of Hedgehog Regulates the Expression of Bone Morphogenetic Proteins to Maintain Germline Stem Cells in Drosophila
Source: PLoS Biol. 2012 Apr 3;10(4):e1001298. doi: 10.1371/journal.pbio.1001298 (PMC3317903; doi:10.1371/journal.pbio.1001298)
Supplement: Table S3 — Average cytoneme length in different experimental conditions. This supplemental table is related to Figure 5. The table shows the average length (in micrometers) of cytonemes that project from wild-type CpCs in wild-type controls and in mosaic germaria containing 1, ≤2, or ≥3 mutant cells. (DOC) [file pbio.1001298.s010.doc]

| **Genotype of niche cells** | | **Cytoneme length ± s. d. (n)** |
| --- | --- | --- |
| Wild-type | All niche cells | 0.93 ± 0.33 (17) |
| *en^-^* CpCs | ≤ 2 cells | 2.06 ± 0.34 (14) |
|  | ≥ 3 cells | 4.45 ± 0.95 (13) |
| *smo^-^* ESCs | = 1 cell | 2.35 ± 0.49 (6) |
|  | ≥ 2 cells | 5.165 ± 1.35 (8) |
| *hop^-^* CpCs | ≤ 2 cells | 1.15 ± 0.32 (12) |
|  | ≥ 3 cells | 1.10 ± 0.35 (8) |
