# Supplementary material for: Cytoneme-Mediated Delivery of Hedgehog Regulates the Expression of Bone Morphogenetic Proteins to Maintain Germline Stem Cells in Drosophila
Source: PLoS Biol. 2012 Apr 3;10(4):e1001298. doi: 10.1371/journal.pbio.1001298 (PMC3317903; doi:10.1371/journal.pbio.1001298)
Supplement: Table S4 — Total cytoneme length per CpC in different experimental conditions. This supplemental table is related to Figure 5. The table shows the average length (in micrometers) of all cytonemes per CpC in wild-type controls and in mosaic germaria containing 1, ≤2, or ≥3 mutant cells. (DOC) [file pbio.1001298.s011.doc]

| **Genotype of niche cells** | | **Total cytoneme length/CpC ± s. d. (n)** |
| --- | --- | --- |
| Wild-type | All niche cells | 1.05 ± 0.37 (15) |
| *en^-^* CpCs | ≤ 2 cells | 2.39 ± 0.61 (12) |
|  | ≥ 3 cells | 5.25 ± 1.31 (11) |
| *smo^-^* ESCs | = 1 cell | 2.82 ± 1.20 (5) |
|  | ≥ 2 cells | 5.90 ± 1.27 (7) |
| *hop^-^* CpCs | ≤ 2 cells | 1.25 ± 0.43 (11) |
|  | ≥ 3 cells | 1.26 ± 0.41 (7) |
